# Multiplex hyperspectral IHC: A 12-plex assay for high-dimensional analysis of the tumor microenvironment

**DOI:** 10.1093/immhor/vlag054

**Published:** 2026-10-15

**Authors:** Kouther Noureddine, Paul Gallagher, John English, Jagoda Korbelik, Anita Carraro, Sylvia Lam, Puneet Arora, Athena Li, Martial Guillaud, Anna McGuire, Calum MacAulay

**Affiliations:** Department of Integrative Oncology, BC Cancer Research Centre, Vancouver, BC, Canada; Department of Integrative Oncology, BC Cancer Research Centre, Vancouver, BC, Canada; Department of Statistics, University of British Columbia, Vancouver, BC, Canada; Department of Integrative Oncology, BC Cancer Research Centre, Vancouver, BC, Canada; Department of Integrative Oncology, BC Cancer Research Centre, Vancouver, BC, Canada; Department of Integrative Oncology, BC Cancer Research Centre, Vancouver, BC, Canada; Department of Integrative Oncology, BC Cancer Research Centre, Vancouver, BC, Canada; Department of Integrative Oncology, BC Cancer Research Centre, Vancouver, BC, Canada; Department of Integrative Oncology, BC Cancer Research Centre, Vancouver, BC, Canada; Vancouver Coastal Health Research Institute, Vancouver General Hospital, Vancouver, BC, Canada; Department of Statistics, University of British Columbia, Vancouver, BC, Canada; Department of Integrative Oncology, BC Cancer Research Centre, Vancouver, BC, Canada; Department of Pathology and Laboratory Medicine, University of British Columbia, Vancouver, BC, Canada

**Keywords:** multiplexed immunohistochemistry, spatial biology, tumor microenvironment

## Abstract

The immune component of the tumor microenvironment (TME) is highly prognostic of patient outcome, further understanding of TME and the spatial organization of the immune cells within the TME is needed for better patient prognosis and treatment planning. Current brightfield immunohistochemistry techniques quantify immune cell counts and density but generally cannot assess the spatial relationship between tumor and immune cells due to the limited number of markers on a single slide. Here, we present a brightfield-based multiplexed immunohistochemistry workflow that combines multiple labels per staining round with iterative cycles of staining, destaining, and hyperspectral imaging to enable high-dimensional immune profiling within a single tissue section. This approach is directly compared with a sequential immunofluorescence technique applied to adjacent tissue sections to compare and evaluate its performance. By integrating serial imaging, sequential labeling, and image registration, we are able to spatially map the TME using standard brightfield microscopy.

## Introduction

The tumor microenvironment (TME) is a highly complex and heterogeneous mixture composed of tumor cells, stroma, and a diverse network of immune cells, and understanding the immune cell interactions and components of this microenvironment is often a key indicator of tumor progression.^1^ The spatial arrangement of the various cellular and structural components within tumor tissue, along with the interactions occurring between cancer cells and their adjacent neighboring cells, crucially influences tumor progression and the effectiveness of therapeutic responses.^2^^,^^3^ Multiple studies have emphasized the significance of specific cell populations within the TME that have been associated with improved outcome in many cancers. This has been shown through examples such as “immunoscore,” which demonstrated that the density of the lymphocyte population CD3/CD8 is a better predictor of survival in colorectal cancer than the TNM (tumor-node-metastasis) stage.^4^ In breast cancer, recent studies of tumor-infiltrating lymphocytes revealed stromal CD3+ T cells and FOXP3+ regulatory T cells were associated with disease-free survival.^5^ In lung adenocarcinoma, analysis of the TME revealed that high-density intratumoral CD8^+^ T cells and CD8^+^ T cells surrounded by tumor cells was significantly associated with nonrecurrence,^6^ highlighting the need for a more detailed and comprehensive analysis of the tumor microenvironment.

Identifying and quantifying the numerous immune cell interactions and cellular populations regulating tumor development requires the use of advanced techniques that permit single-cell profiling. However, a major limitation of such high-throughput analytical methods is tumor tissue availability.^7^ Although such methods offer insight into the heterogeneity and state of individual cells within the tissue, they require disruption of the tissue and loss of the spatial organization of the cells.^8^^,^^9^ Given the importance of understanding how different cells interact at the TME level, technologies that preserve the spatial context of cells are essential. Multiplex immunohistochemistry (IHC) is one such method that offers critical insight into a patient’s cancer by characterizing the tumor biology, permitting the simultaneous detection of multiple protein biomarkers on the same tissue section while preserving the tissue.^10^ In the past few years, there has been an emergence of multiplexed immunostaining methods that permit the high-dimensional analysis of complex cell populations on a tissue. However, many of these methods require the use of fluorescent dyes associated with costly equipment and reagents.^11^^,^^12^ Recent advances in chromogenic-based IHC methods have enabled characterization of several parameters on a single tissue section.^7^^,^  ^13–15^ These methods rely either on iterative cycles of staining and destaining of a single chromogen (e.g. AEC) across multiple rounds, or the use of covalently deposited chromogens with relatively narrow absorbance spectra.^15^ While these approaches enable multiplexing, they are limited by increasing staining cycles or the need for specialized chromogens that may not be compatible with routine workflows.

To address the need for high-dimensional analysis of tissue lesions in clinical pathology in a shorter time frame, we have developed a multiplexed brightfield IHC procedure that combines multiple labels per round over several sequential rounds. This technique, termed multiplexed hyperspectral IHC (MH-IHC), integrates serial imaging, sequential labeling and hyperspectral brightfield imaging and can be easily applied on any formalin-fixed, paraffin-embedded (FFPE) tissue to achieve up to 7 markers on a single tissue section in just 2 rounds of staining. MH-IHC utilizes standard chromogens and leverages hyperspectral imaging and destaining to perform unmixing of chromogens with overlapping spectral signatures. This enables simultaneous detection of multiple chromogens per round over multiple rounds, increasing the number of markers achievable on a single slide.

Through destaining multiple chromogens in one pass, rather than in a single chromogen, and by utilizing multispectral scanning, MH-IHC permits the detection of IHC-linked chromogens that may be difficult to resolve by human eye due to different staining densities or colocalized staining of the same structure. As with most other methodologies, utilizing image registration enables the detection of larger panels using adjacent sections while preserving tissue architecture and subjecting the tissue to a minimal number of washes. A comparison of the proposed approach with common existing multiplexed brightfield IHC methods is summarized in Table 1.

**Table 1.** Comparative summary of various multiplexed brightfield IHC methods enabling detection of multiple biomarkers on a single tissue slide.

Feature	MH-IHC	**MICCSS** ^13^	**Multispectral brightfield** ^15^
**Multiplexing method**	Iterative staining of multiple antibodies per round	Iterative staining of a single antibody per round	Staining of multiple antibodies in 1 round only
**Standard chromogens**	Yes	Yes	No
**Chromogens per cycle**	3 or 4 per cycle	1 per cycle	4 or 5 per cycle
**Chromogen destaining**	Yes	Yes	No
**Cycles to achieve 7**	2	7	1
**Markers on a single slide**	7	7	4 or 5
**Fixed Spectra**	No	Yes	Yes
**Unmixing method**	MCR-ALS	Not required	Non-negative least square
**Imaging method**	Hyperspectral Imaging	Standard RGB Imaging	Multispectral Imaging

Here, we describe the MH-IHC workflow and its advantages in evaluating cell types requiring multiple markers to identify, such as those in the complex TME.

## Methods

### Patient tissue accrual

FFPE tonsil, colon, and lung tumor tissue was obtained from the Tumor Tissue Repository of the British Columbia Cancer Agency or Vancouver General Hospital under informed written patient consent and with approval from the University of British Columbia–BC Cancer Agency Research Ethics Board (H23-03298).

For analysis of early-stage non-small cell lung cancer (eNSCLC), a total of 18 treatment-naïve patients with e NSCLC who underwent surgical resection for curative intent were included in this study. Recurrence cases were defined as patients diagnosed with NSCLC who had recurrence within 5 yr after surgery. Control subjects were defined as patients with at least 5 yr of postsurgical follow-up who had no evidence of recurrence. Patient demographic information is presented in Table S1. Informed consent was obtained from all study participants.

### Immunohistochemistry

#### Deparaffinization and staining

FFPE tissue sections (5 μm) were placed for 30 min in a 60 °C oven before following the deparaffinization protocol. The deparaffinization and rehydration protocol entails incubation in xylene twice for 5 min each, followed by incubation in 100% ethanol for 20 min.

Antigen retrieval was performed using the Decloaking Chamber Plus (Biocare Medical). Slides were submerged in diva decloaker solution (DV2004LX; Biocare) and heated to 110 °C for 30 min. Following antigen retrieval, slides were removed from the decloaking chamber and rinsed thoroughly with distilled water (dH2O). To prepare slides for staining, tissue sections were surrounded by a hydrophobic barrier using a PAP pen and loaded on to the IntelliPATH FLX automated stainer (Biocare Medical), which is capable of processing up to 50 slides simultaneously.

Our protocol requires a single tissue section to be subjected to multiple rounds of staining. After some experimentation, we found that an adequate process is as follows. In the first round, slides are blocked with peroxidazed-1 (PX968H; Biocare) and background sniper 1 (BS966H; Biocare) for 5 and 10 min, respectively. In this example, slides were then stained with anti-PD1 (ab52587, clone NAT105; Abcam) diluted in DaVinci Green diluent (Biocare) for 30 min, followed by an AP conjugated secondary antibody (MACH 2 MS-AP, MALP521G; Biocare) for another 30 min, the secondary antibody was revealed using Ferangi Blue chromogen (FB813H; Biocare) for 7 min. Slides were then rinsed with dH_2_O, incubated in SDS-glycine pH 2.0 for 45 min at 50 °C, and rinsed with dH_2_O.^16^ Slides were then stained with an anti-CD8 (ab17147, clone C8/144B; Abcam)/anti-PDL1 (ab228462, clone SP142; Abcam) cocktail for 30 min, followed by MACH 2 Double Stain 1 (MS-AP/RB-HRP, MRCT523G; Biocare) secondary antibody, and finally Warp Red and AEC (ab64252; Abcam) chromogens were applied for 7 and 15 min, respectively, before adding the hematoxylin (S3301; Agilent) counterstain. Slides were then removed from the stainer and mounted with an aqueous mounting medium (Abcam, ab128982). After multispectral scanning the slides, coverslips were removed in a hot water bath (50 °C) and tissue sections were destained in organic solvent (50% ethanol, 15 min; 100% ethanol, 15 min; 100% xylene; 30 min, 100% ethanol, 15 min; and 50% ethanol, 15 min). This step removed chromogen staining only. To remove all the primary-secondary antibody pairs from the first round of staining, slides were placed in an SDS glycine solution for 45 min at 50 °C. Slides were then washed with dH_2_O and ready for next round of staining. For the second round, after loading the slides into the stainer, the slides were blocked with peroxidazed-1 (Biocare) and background sniper 1 (Biocare) for 5 and 10 min, respectively. Slides were then stained with an anti-FOXP3 (ab99963, clone SP97; Abcam)/anti-KI67 (sc23900; Santacruz) cocktail for 30 min, followed by MACH 2 Double Stain 2 (MS-HRP/RB-AP, MRCT525G; Biocare) secondary antibody, and finally Warp Red and DAB (DB801L; Biocare) chromogens were used as the chromogenic substrates, respectively. Next, slides were washed in SDS glycine solution for 45 min at 50 °C and an anti-CD20 (ab78237, clone EP459Y; Abcam)/anti-CAS3(3CSP01; Thermo Fisher Scientific) cocktail was added to the slides before adding MACH 2 Double Stain 1 secondary antibody. Finally, AEC and Ferangi Blue (FB813H; Biocare) chromogens were added to visualize the respective antibodies, followed by hematoxylin counterstain. For an adjacent tissue section, a similar protocol was applied using primary antibody COL1 (ab34710; Abcam) visualized with AEC. Following COL1 staining, the slide was imaged, destained, and subjected to antibody elution to enable a subsequent round of multiplex chromogenic staining. In the second round, Elastin (ab23747; Abcam) was visualized with AEC, vimentin (ab92547; Abcam) with Ferangi Blue, CD31 (ab28364; Abcam) with Warp Pink and pan-cytokeratin (ab80826, clone AE1/AE3; Abcam) with DAB. The primary antibodies and their respective working dilutions are summarized in Table S2. Antibody staining and background were assessed using known positive tissue controls, negative controls, no-primary-antibody controls, and single-plex controls.

### Destaining and antibody elution

#### Destaining

Slides were destained in several washes of alcohol and xylene. After imaging of the first round of staining, slides must be destained and processed for the next round of antibodies. To gently remove the coverslip without causing any damage to the tissue, slides are immersed in a hot water bath (50 °C) for 10 to 15 min. Once the coverslip has been removed, the slides must be washed in a cold water to clean off any remaining aqueous mounting medium. To remove all the first-round chromogens (AEC, Warp Red, and Ferangi Blue), slides are immersed in 50% ethanol for 15 min, followed by 15 min in 100% ethanol and 30 min in xylene. After incubating the slides in xylene, they are placed one last time in 100% ethanol for 15 min followed by 50% ethanol in 15 min to completely remove any of the remaining chromogens (Fig. 1).

**Figure 1.**
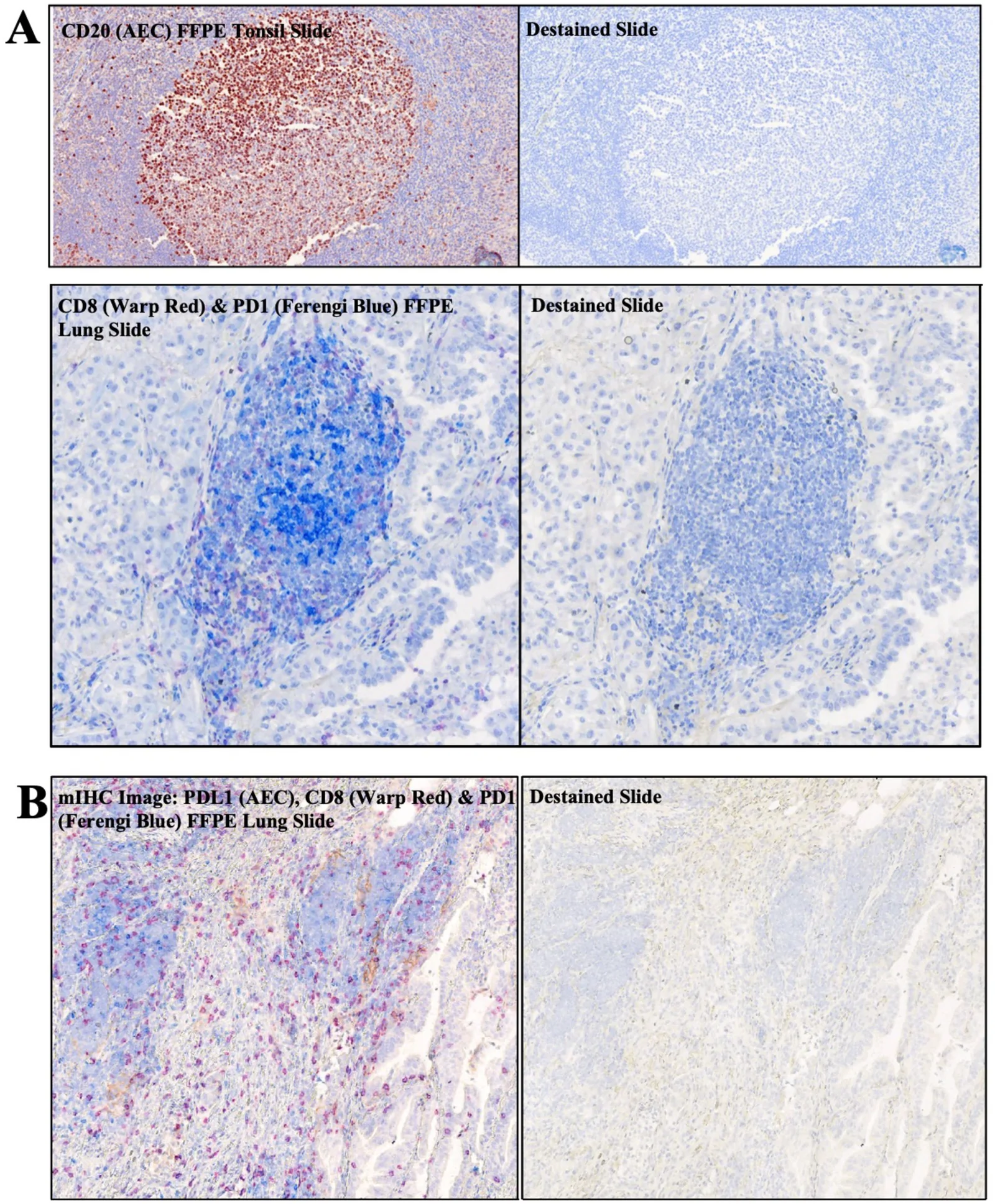
(A) FFPE tonsil and lung tissue sections were stained with primary antibodies to demonstrate the success of destaining chromogens AEC, Warp Red, and Ferangi Blue. (B) FFPE lung tissue stained with 3 primary antibodies followed by chromogens AEC, Ferangi Blue, and Warp Red in a single staining round to demonstrate the success of destaining 3 chromogens at once. AEC produces a red precipitate, Warp Red produces a pink precipitate, and Ferangi Blue produces a blue precipitate.

#### Antibody elution

After the slides have been destained, to prepare the tissue slide for the next round of antibodies, any remaining antibodies must be removed.^16^ SDS glycine pH2 buffer solution was prepared by mixing 0.94 g glycine in 25 mL 20% SDS and increasing the volume to 500 mL with distilled water. Once the distilled water is added, the pH is adjusted to 2 by adding HCL. The prepared solution is then placed in a jar and preheated to 50 °C before adding the slides and incubating them in the solution at 50 °C for 45 min. After elution, slides are washed thoroughly with distilled water making them ready to be processed for the next staining round.

During coverslip removal, slides were immersed in distilled water and the coverslips were allowed to detach without the application of mechanical force, thereby minimizing the risk of tissue displacement or damage. Tissue sections were mounted on positively charged glass slides to promote tissue adherence and minimize tissue loss during repeated staining, antibody stripping, and coverslip removal. The solvent incubation and antibody-stripping conditions used in this study have been validated over a greater number of sequential staining rounds, with minimal tissue loss or morphological damage observed. Furthermore, limiting the present protocol to the minimum number of staining cycles required for multiplexing reduced repeated tissue processing and the associated risk of tissue deformation, loss, or registration errors.

### Multiplex immunofluorescence

Multiplex immunofluorescence (mIF) was done on the Lunaphore COMET 1.0 platform which is an automated stainer that enables simultaneous detection of multiple markers on the same tissue section by performing iterative cycles of staining, imaging and elution. FFPE tissue sections of human lung samples were stained and imaged on the COMET with a high-plex assay consisting of 19 markers. The markers selected included 13 markers from Lunaphore’s prevalidated immune-oncology SPYRE-Kit plus an additional 6 markers: COL1 (ab138492; Abcam), elastin (ab213720; Abcam), cytokeratin (ab80826; Abcam), caspase-3 (3CSP01; Thermo Fisher Scientific), CD31 (ab28364; Abcam), and vimentin (ab92547; Abcam). Prior to insertion into the Lunaphore system for staining, dewaxing and antigen retrieval is done on the PT module (Epredia; A80400012) with Dewax and Hier retrieval buffer pH6 solution (Lunaphore).

### Tissue imaging

#### Hyperspectral imaging

For imaging the chromogenic IHC markers, our in-house-developed automated hyperspectral whole slide imaging system was used to image the slides after each round of staining. The hyperspectral scanner consists of a brightfield microscope (Zeiss Axioscope 2 Mot plus microscope and Zeiss 20X [NA 0.75] plan APROCHROMAT objective lens) and camera (1920 × 1200 pixel 160 frames per second, GS3-U3-23S6M-C FLIR grasshopper 3 USB 3.0 monochrome camera) with a computer-controlled x-y stage (Marzhauser Wetzlar SCAN series), in which the condenser has been replaced by an in-house, inexpensive (∼$150) 7-LED illumination light source (Fig. S1). Images were acquired with a pixel sampling spacing of 0.233 μm (20× magnification), and the area to be imaged is selected by the user prior to imaging. Spectral images from a blank area of each of the slides were used for flatfield calibration purposes across the spectral range imaged. The system automatically sequentially imaged and collected (using a continuously active focus) up to 7 different spectral images (nominal center wavelengths 407 nm, 460 nm, 523 nm, 590 nm, 623 nm, 660 nm, 740 nm; the actual LED peak wavelengths were measured to be 402 nm, 458 nm, 518 nm, 584 nm, 623 nm, 647 nm, and 703 nm) per camera field. The LEDs were triggered in sequence (controlled by the camera sync pulse) with a dark image added to define the end of the sequence. This allowed the camera to run at 160 images per second, resulting in 20 hyperspectral 7 wavelength images per second. Once the area was imaged using a grid of tiled camera fields, the system stitched all overlapping camera fields together to make a seamless hyperspectral image. Other hyperspectral commercial bright field microscopy systems could also be used to image the slides such as with the Mantra or PhenoImager HT produced by Akoya Biosciences.

#### Spectra unmixing

Using the absorption spectra of each chromogen and hematoxylin, the stains were computationally unmixed to determine the concentration of each stain for every pixel in the selected area. For spectral unmixing, reference absorption spectra were obtained either empirically from the acquired images or from predefined reference spectra, depending on which approach provided optimal chromogen separation. For image-derived spectra, regions containing spectrally pure staining for individual chromogens were manually selected to extract representative absorption profiles. Predefined absorption spectra were used for chromogens with well-characterized and reproducible spectral signatures. The selected reference spectra were then used for spectral unmixing of the multiplexed images. The choice of reference spectrum for each chromogen was based on qualitative assessment of unmixing performance and minimization of spectral crosstalk. The spectral unmixing assumes that every pixel in the flat field and dark field corrected recorded images (7 wavelengths) are a linear combination of the concentration of the individual stains, C, occurring at that pixel, D, weighted by the absorption characteristics, S, of each of the stains occurring at that pixel. When mathematically separating the components, it adds an additional error term, E, to each pixel to compensate for the electronic and photonic noise in the images.


(Equation 1) D=CS+E

*D = Data Matrix* (log-transformed flat field and dark field corrected multispectral images)

*C = Concentration Matrix (per pixel)*

*S = Pure Spectra (per multispectral wavelength)*

*E = Error Term*

For unmixing, a Multivariate Curve Resolution—Alternating Least Squares algorithm (MATLAB R2014a; The MathWorks) was used to separate the linear combinations for the absorption stains, each varying in concentration at each individual pixel. The analysis involved transforming the collected hyperspectral image data using a logarithm base 10 scale to make the result linear with amount of absorption occurring at each pixel (Beer-Lambert law is commonly applied to chemical analysis measurements to determine the concentration of chemical species that absorb light).^6^^,^^17^ These data were then modeled to represent concentration images for each stain, each multiplied by the corresponding stain spectra plus an error term.^18^^,^^19^ To use this method, several assumptions were made: (i) the spectra remained constant, (ii) there were no negative concentrations, and (iii) the sum of concentrations at each pixel did not necessarily have to total one, a condition referred to as additivity. By adopting these assumptions, the need to previously know the absorption spectra of the glass, and tissue was obviated, which would have been necessary if the additivity constraint had been imposed. The result of the unmixing were concentration images (bright object representing a high concentration on a dark background of low concentration), which appear identical to fluorescence images and were treated as such.

### Image analysis

After all images had been acquired from adjacent tissue sections, image alignment was preformed predominantly using Visiopharm software.^20^

#### Image alignment

Images obtained from the same slide but acquired across different staining rounds were first aligned using the Tissue Align module in Visiopharm. Next, these aligned images from the from the two adjacent slides were aligned (Fig. S2). Once all images had been aligned, the registered images were exported and combined into a single OME.tiff containing 13 channels (hematoxylin nuclear counterstain and 12 biomarkers) using ImageJ (version 1.54f, National Institutes of Health).

#### Image analysis

Segmentation and phenotyping were done in Visiopharm, equipped with a pretrained deep neural network for nuclei segmentation, it was further trained (tuned) for more precise segmentation of the hyperspectral unmixed hematoxylin images. Following image alignment, the combined OME.tiff file containing all channels was loaded into Visiopharm’s image analysis module and using the Nuclei Detection APP (Nuclear Detection, AI [Fluorescence #10169]), the hematoxylin concentration image was segmented. Specifically, the nuclei segmentation APP was further trained using hematoxylin-stained concentration images to reduce the amount of segmentation errors (nuclei the APP failed to recognize/segment).

After successful segmentation, utilizing Visiopharm’s phenoplex workflow, we could verify and quantify the cellular phenotypes present in each image. This step involved reviewing the staining for each individual marker and selecting appropriate intensity thresholds for all markers. Following thresholding, the Phenoplex workflow identified all possible marker combinations present within each image.

## Results

### MH-IHC integrates sequential labeling with serial imaging to facilitate spatial characterization of the TME

In the past, conventional imaging methods used with IHC and IF were limited to visualizing only a small number of biomarkers simultaneously. However, recent advances in imaging and analytical tools have significantly advanced our capabilities to study the TME by overcoming these limitations.^21^^,^^22^ For brightfield IHC, because of the limit on the number of chromogens that can be used in parallel on a single tissue slide, multiplex IHC (mIHC) methods that rely on consecutive cycles of staining and destaining of a single chromogen (AEC) over repetitive rounds proved to be crucial in studying the complexity of the TME.^7^^,^^13^^,^^14^ However, we sought to determine whether combining multiple antibody labels in a single staining cycle, using 3 distinct chromogens that can be destained and accurately unmixed, could further characterize the TME in a much shorter time frame, overcoming limitations associated with mIHC (Fig. 2).

**Figure 2.**
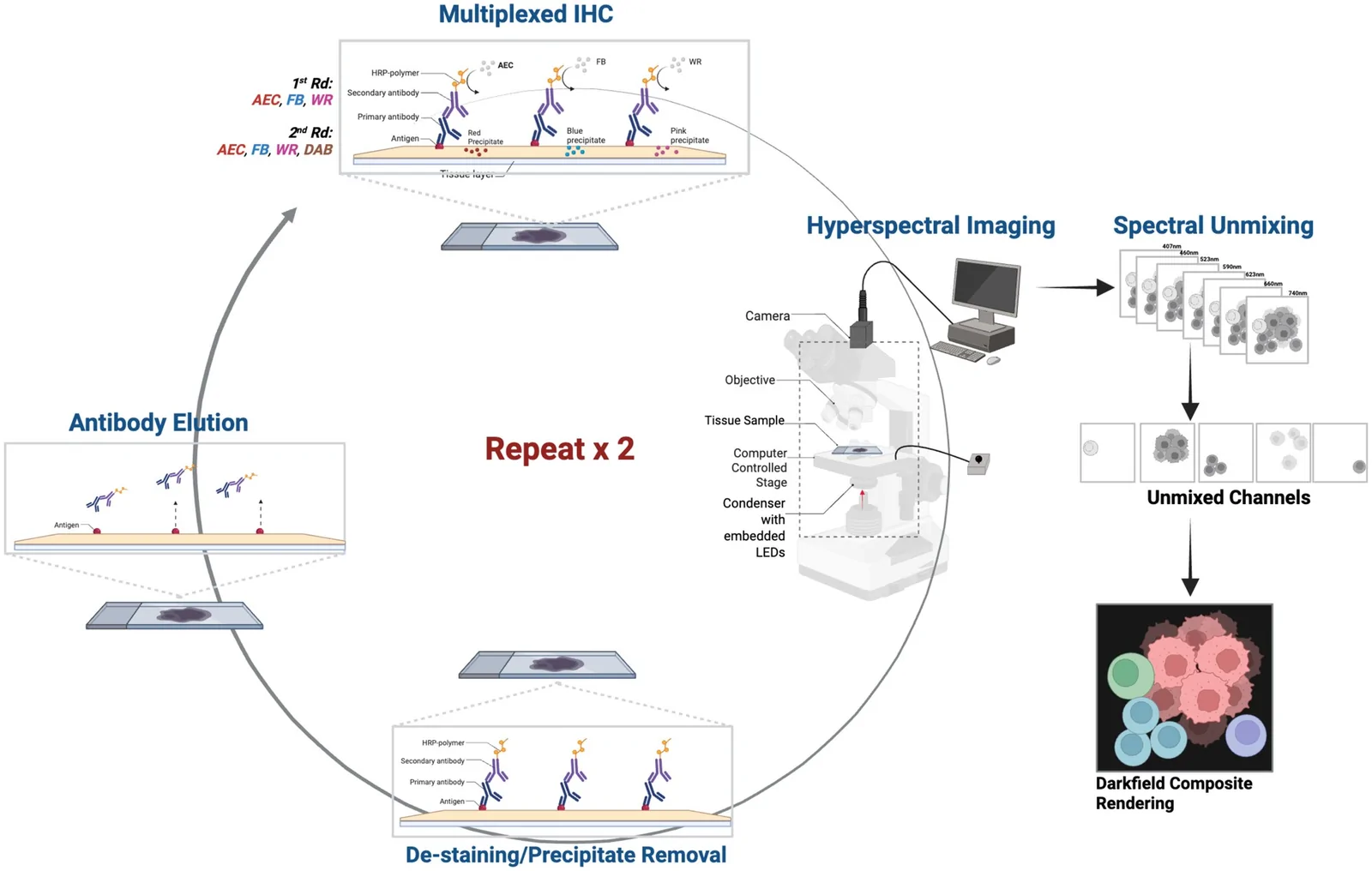
MH-IHC workflow. FFPE tissue section is stained in the first round with primary antibodies, followed by secondary antibodies conjugated to either horseradish peroxidase or alkaline phosphatase, and finally the 3 washable chromogens. Once slides are stained, they are scanned using our in-house hyperspectral scanner. After scanning, the coverslip is removed, and slides are destained in several washes of ethanol and xylene. Prior to restaining, slides are incubated in an antibody elution solution to remove any bound primary, preparing the tissue for a new round of staining. FB, Ferangi Blue; Rd, round; WR, Warp Red, Created with BioRender.com.

To determine the maximum number of chromogens, we could combine in a single staining round of IHC, we attempted to test how many available chromogens could be successfully removed using a published destaining protocol used to destain AEC chromogen.^7^ We stained several FFPE tissue slides with AEC and a separate tissue slide with chromogens Warp Red and Ferangi Blue (Fig. 1A). After immersing the slides in alcohol and xylene under the same incubation times used to wash off AEC, we found that both chromogens, Warp Red and Ferangi Blue, began to slightly fade, implying that they could be destained. Upon increasing the incubation time in alcohol and xylene, we were able to successfully remove both blue and pink chromogens in addition to the AEC chromogen (Fig. 1A), therefore allowing us to combine the 3 chromogens in parallel on the same tissue slide and limiting the need for multiple rounds of staining (Fig. 1B). DAB chromogen could not be destained, thus limiting its inclusion to the final round of IHC staining.

Given our success in destaining multiple chromogens in a single staining round, we asked whether combining consecutive cycles of mIHC staining with hyperspectral scanning could further enhance our understanding of the interactions occurring within the TME by increasing the number of markers we could achieve on a single tissue section. Thus, a single FFPE tissue slide was first stained with a set of 3 primary antibodies, followed by its respective secondary antibody and finally a horseradish peroxidase– or alkaline phosphatase–conjugated chromogen. The absorption-based chromogens AEC, Warp Red, and Ferangi Blue were used to reveal each antibody. After staining, slides were mounted and hyperspectral imaged using 7 different wavelengths to allow us to accurately spectrally unmix each antibody using its respective absorption spectra (Fig. 3). To prepare the slides for a second cycle of staining, the colored precipitates produced from each of the 3 chromogens were removed by immersing the slides in several concentrations of alcohol and xylene. Once the slide has been successfully destained, any remaining antibodies from the first round of staining were removed by placing the slides in an SDS glycine buffer solution to prevent any remnant reactivity to primary or secondary antibodies used in the first cycle.^16^ In the final round of staining, an additional chromogen, DAB, can be added to achieve a total of 4 antibody labels in a round. This allowed us to achieve up to 7 antibody labels on a single tissue slide in just 2 cycles of staining (Fig. 2).

**Figure 3.**
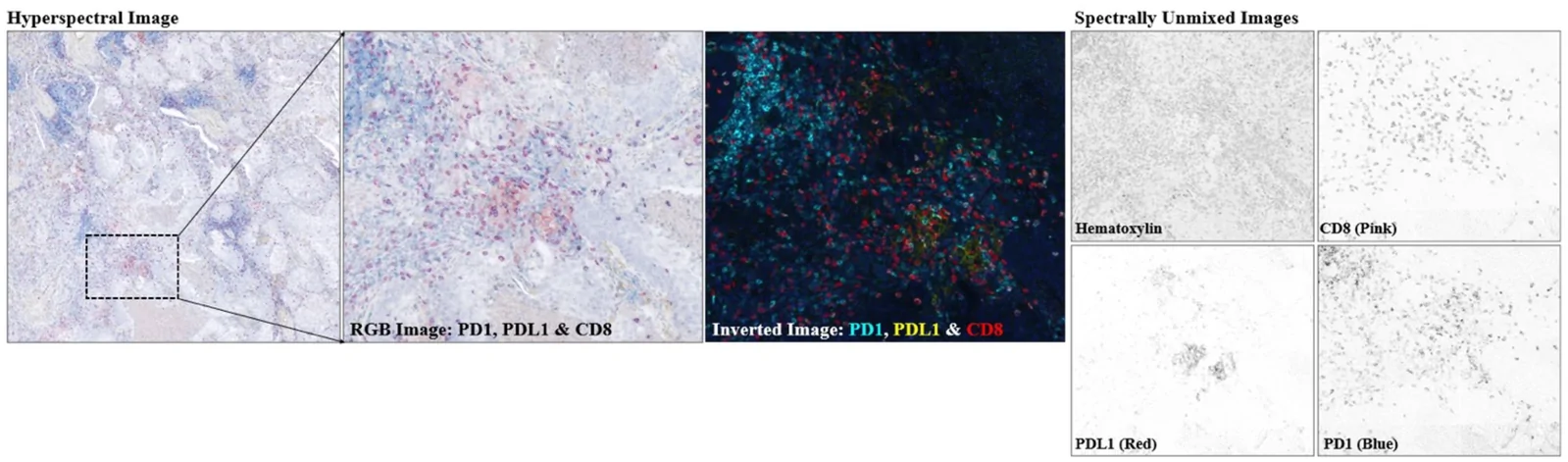
mIHC FFPE tissue stained with 3 chromogens and hyperspectral imaged. Using the hyperspectral image data, images are spectrally unmixed to generate individual concentration images corresponding to each chromogen (center). To allow for better visualization of multiple markers (colocalization on a single cell), we inverted the data to generate absorption-like images (right).

### Hyperspectral imaging enables unmixing of spectrally overlapping signatures with minimal spectral crosstalk

Given that conventional chromogens widely used in routine pathology laboratories exhibit broad and often overlapping spectral profiles (Fig. 4), we evaluated the potential for spectral crosstalk between chromogens, with particular focus on chromogens exhibiting similar spectral characteristics (e.g. Pink and AEC [red]). To address this, FFPE tonsil tissue sections were stained with only a single chromogen and unmixed using the full spectral model (hematoxylin, pink, red, blue, and DAB). In each case, the signal was predominantly confined to the expected channel, with minimal bleed-through observed in off-target channels (Fig. 5).

**Figure 4.**
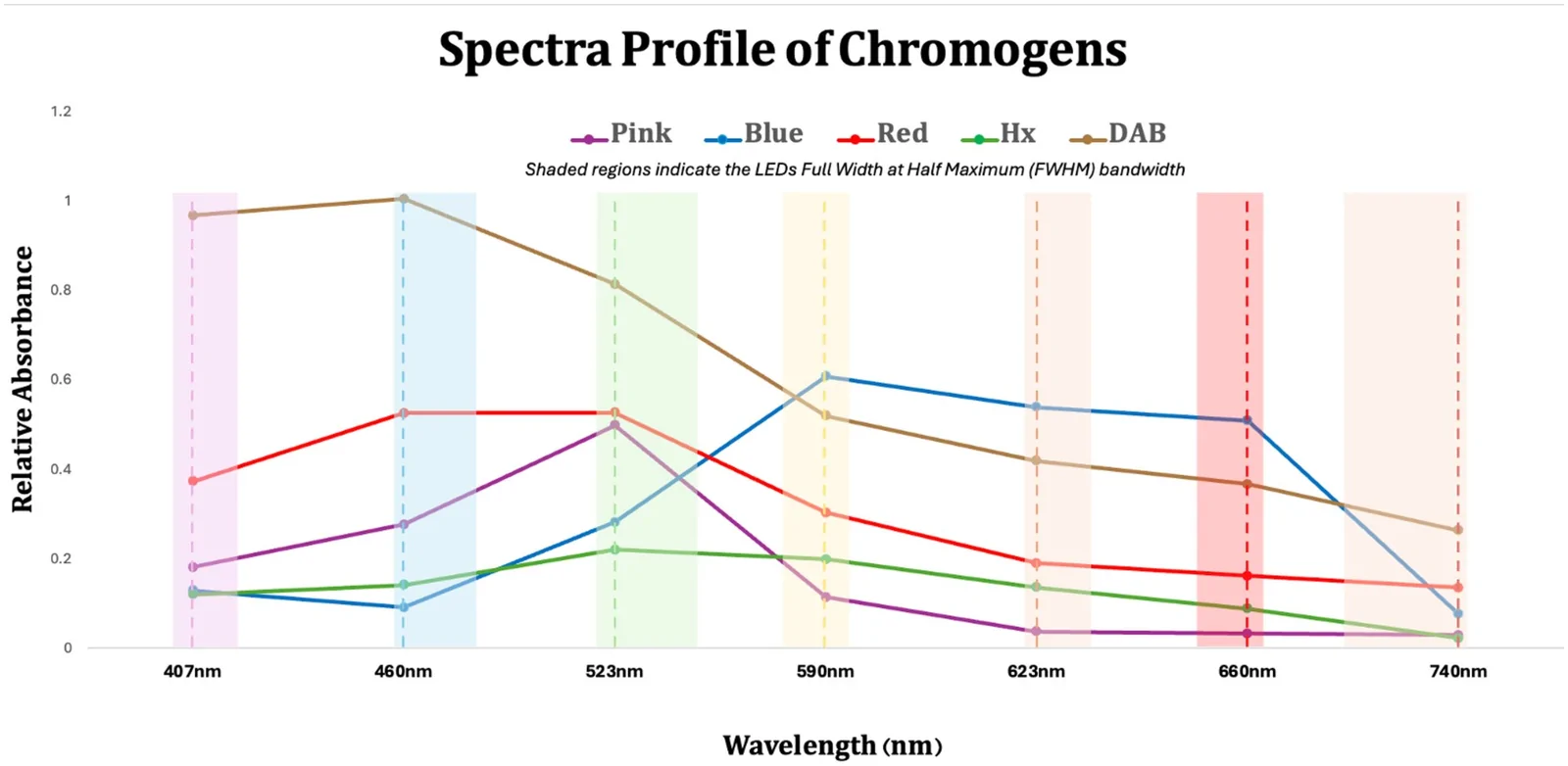
Spectral profile of chromogens. Relative absorbance of chromogens measured at the 7 LED illumination wavelengths, overlaid with the full width at half maximum bandwidths of the LED illumination channels.

**Figure 5.**
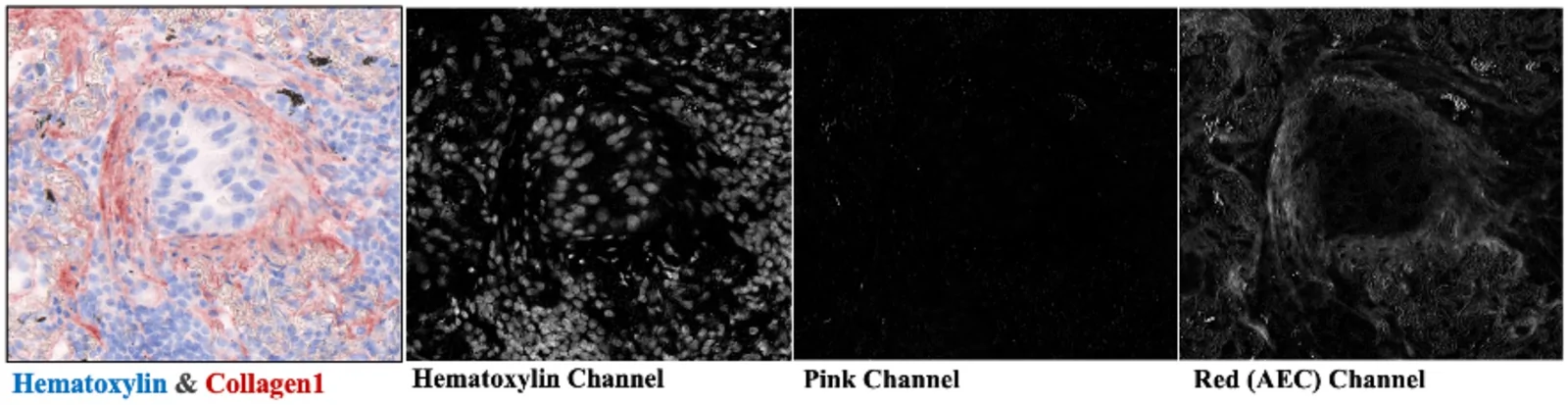
Spectral unmixing of single-stained images. Representative image of a FFPE tonsil tissue section stained with type 1 collagen with AEC chromogen and the corresponding unmixed images generated using the full spectral model. Despite AEC (red) and Warp Pink chromogens having the most overlapping spectral profiles, spectral unmixing enables separation of their signals into distinct channels across with minimal bleed-through, which in the pink channel corresponds to mostly the black debris in the image whose spectral contribution was not explicitly encompassed in the unmixing.

Furthermore, despite the evident significant spectral overlap in the absorbance images prior to unmixing (Fig. 6A), multiplex-stained samples demonstrated effective separation of colocalized chromogens, supporting the robustness of the unmixing approach. These results indicate that spectral unmixing effectively controls crosstalk and enables accurate separation of overlapping chromogen signals (Fig. 6B).

**Figure 6.**
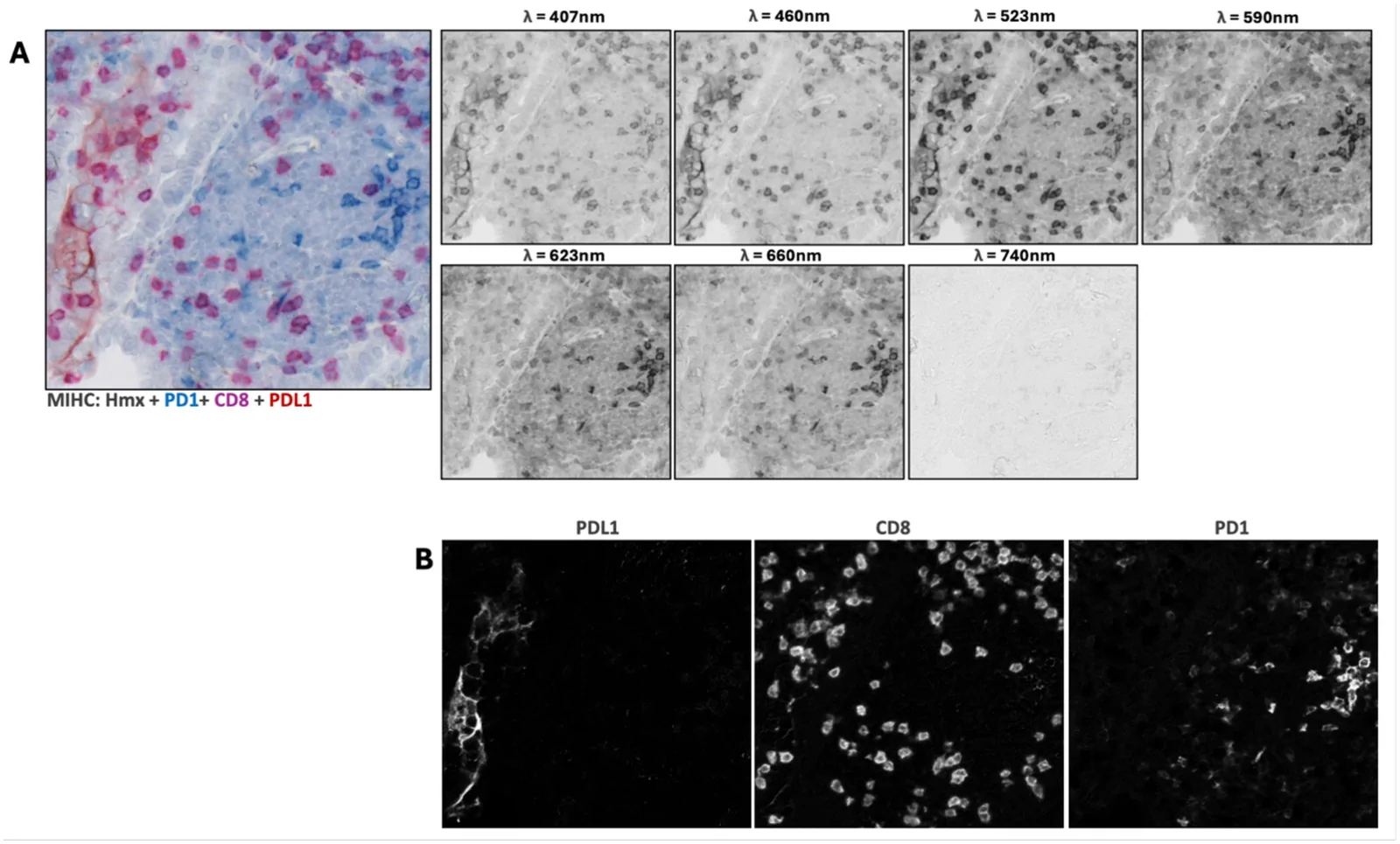
mIHC applied to NSCLC tissue section demonstrating chromogen separation. (A) Images acquired at each wavelength. (B) Corresponding unmixed images for each chromogen following spectral unmixing, illustrating effective separation of colocalized signals.

### MH-IHC preserves tissue integrity

One potential issue with repeatedly staining and destaining a tissue multiple times over several rounds, is the risk of altering the tissue’s structure and antigen expression. To address this, we performed a series of experiments on tonsil tissue to determine the optimal number of staining cycles that can be achieved without observing tissue degradation.

We stained/destained FFPE tonsil and colon tissue with a single antibody over several rounds under similar conditions, observing no tissue loss even after 4 rounds. In Figure 7, we can see the results of FFPE colon tissue section stained/destained over several rounds using only AEC. Indicating that the process of staining, destaining, coverslip removal, and antibody stripping causes very little to no tissue loss or damage. To further examine whether staining multiple biomarkers simultaneously can impact labeling efficiency or tissue integrity, we performed multiple rounds of multiplex staining on FFPE tonsil and lung tissue sections, applying 3 antibodies in each round, to establish the limit of multiplex rounds achievable before tissue degradation is observed. Our findings indicated that two rounds of multiplex staining on a single slide yielded optimal staining results. While a third round of staining was attainable and yielded accurate staining, the consistency of results varied across various samples and experiments. For this reason, we decided to limit the number of staining rounds to two, subjecting the tissue to a minimum number of washing steps.

**Figure 7.**
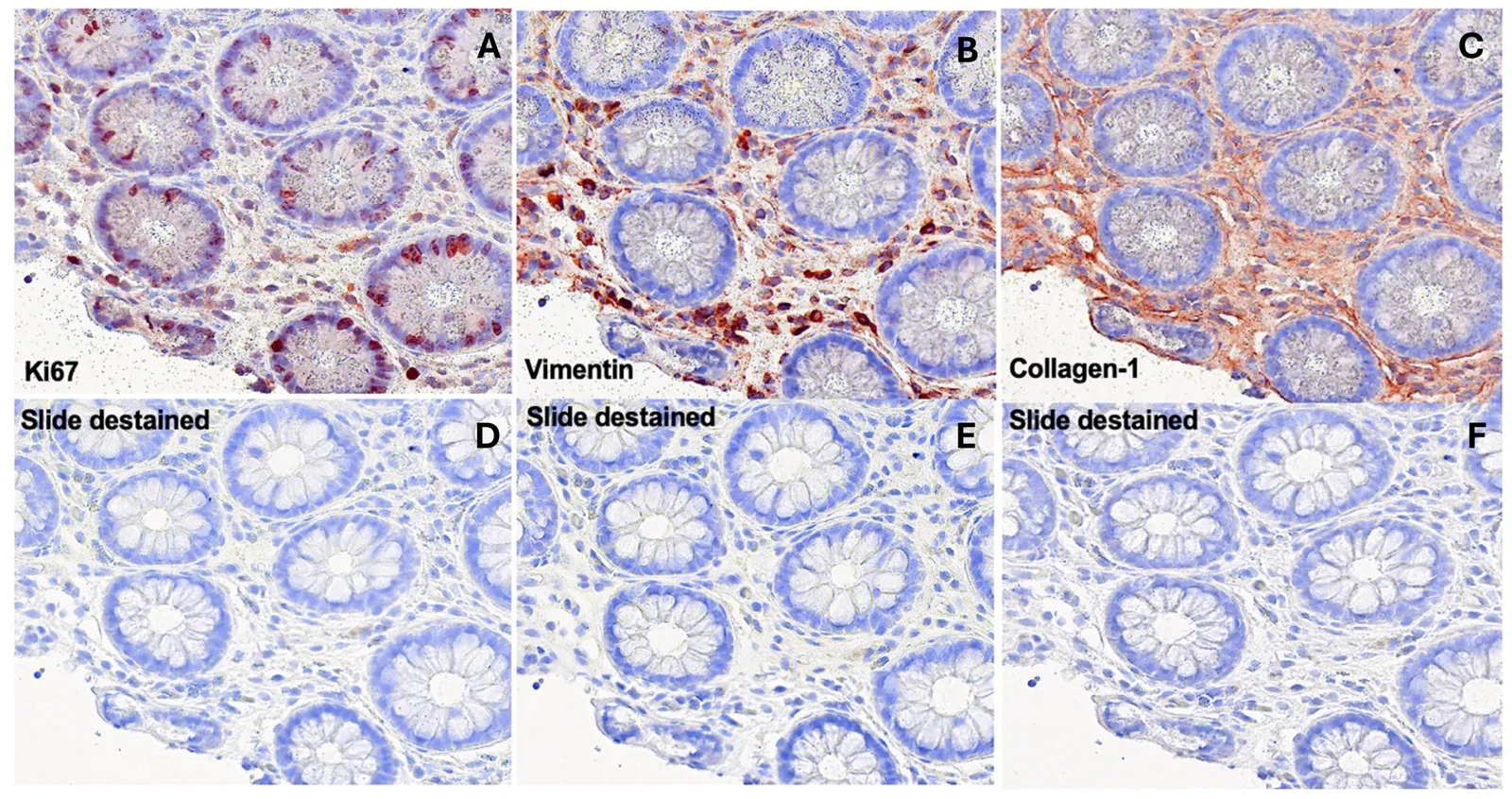
FFPE colon tissue slide stained/destained over several rounds with AEC chromogen. (A) FFPE colon slide stained with Ki-67 using AEC chromogen in the first round of IHC staining. (B) After destaining, the same slide is subjected to a second round of staining with vimentin using AEC chromogen. (C) The third round of IHC staining on the same tissue slide, collagen1 is applied to the tissue followed by AEC chromogen. (D) FFPE tissue slide destained and imaged after one round of IHC. (E) FFPE tissue slide destained and imaged after a second round of IHC. (F) FFPE tissue slide destained and imaged after a third round of IHC.

### MH-IHC provides an accurate spatial representation of the tissue while preserving antigen expression

By combining multiple rounds of staining on a single tissue section, we can analyze the cell-cell interactions and spatial relations between tumor and immune cells in the TME. However, one potential concern when performing multiplex staining and subjecting the tissue to repetitive rounds of staining/destaining is the accuracy of the multiplex imaging data generated, including whether the binding specificity of the antibody is impacted. Particularly, when two or more biomarkers are expressed on the same cell type. To address this, we compared the proportion of positive cells for a given marker in the second round of mIHC to its corresponding single-plex image. We stained serial FFPE tonsil tissue sections with a single biomarker and 1 FFPE tonsil slide with the MH-IHC protocol, combing the 3 markers used in the single-plex staining in the second round of the protocol (Fig. 8). After staining, slides were imaged and the mIHC images were spectrally unmixed to accurately separate individual chromogenic signals, enabling quantification of the percentage of positive cells for each biomarker. Cell detection and classification were performed using QuPath, which using marker-intensity thresholds is able to detect the percentage of positive cells for each specific marker. After quantifying each marker, we observed minimal differences in the proportion of positive cells expressed in the single-plex slides versus multiplex slide after 1 round of destaining (Fig. 9). Suggesting MH-IHC provides an accurate representation of the TME and preserves antigen expression.

**Figure 8.**
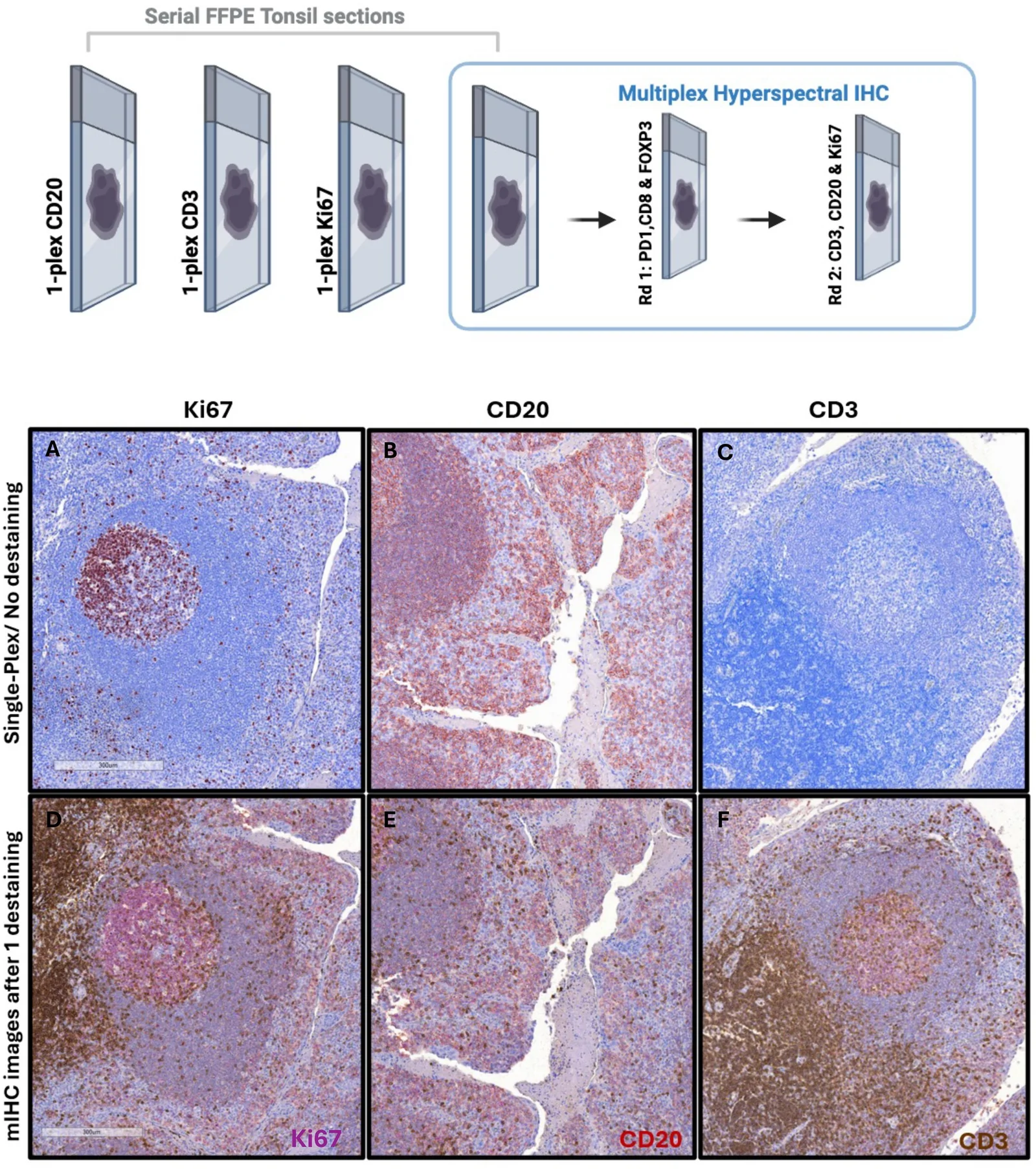
Schematic of the validation workflow used to compare marker detection between conventional single-plex IHC and MH-IHC. Three serial FFPE tonsil sections were stained individually with CD20, CD3, or Ki67 using standard single-plex IHC. A fourth serial section underwent the MH-IHC workflow, consisting of an initial round of immunostaining followed by chromogen destaining and antibody elution before a second staining round incorporating CD20, Ki-67, and CD3. Positive cell counts obtained from the multiplexed section were compared with those from the corresponding single-plex stained sections to assess marker preservation and staining performance following iterative MH-IHC processing. (A) FFPE tonsil section stained for Ki-67 and visualized using AEC chromogen. (B) FFPE tonsil section stained for CD20 and visualized using AEC chromogen. (C) FFPE tonsil section stained for CD3 and visualized using Ferangi Blue chromogen. (D–F) FFPE tonsil sections stained using the MH-IHC workflow and used for comparison with corresponding single-plex stains. Ki67 (Warp Red, pink), CD20 (AEC, red), and CD3 (DAB, brown). Scale bars = 300 μm.

**Figure 9.**
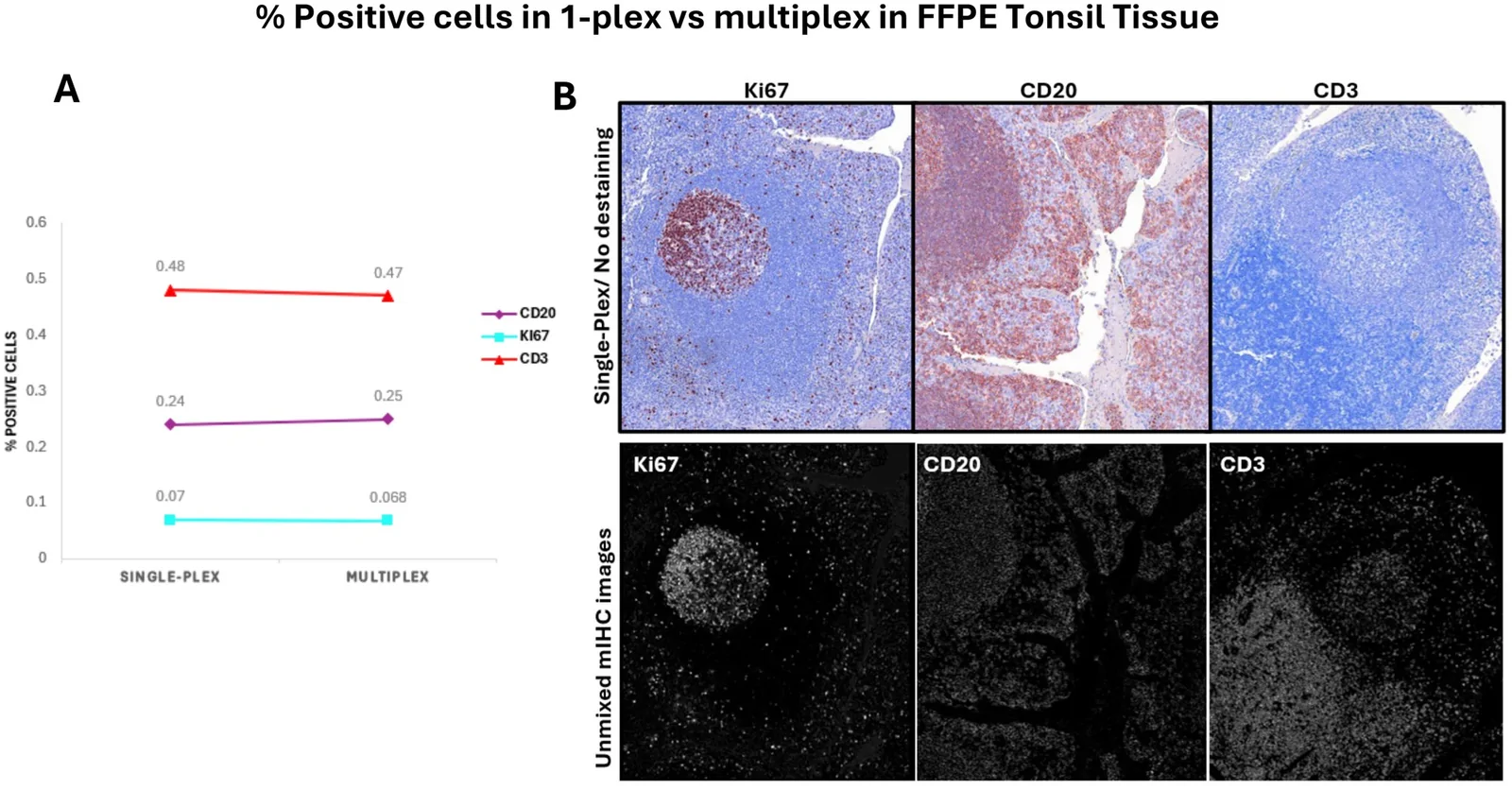
(A) Percent positive cells for markers CD20, CD3, and Ki-67 in both single-plex and multiplex image. (B) Single-plex IHC images coupled with their respective spectrally unmixed image of FFPE tonsil tissue slides stained with a single primary antibody followed by AEC chromogen.

### MH-IHC staining results correlate with mIF

To further assess and validate our mIHC staining results, we compared them to marker-based cell counts from mIF with staining performed by the Lunaphore COMET 1.0 system. Validation was performed using 5 pairs of adjacent serial FFPE lung tissue sections to assess concordance in marker distribution and spatial localization. For the mIF analysis sections were stained using an identical panel of 12 markers. For direct comparison, a single region of interest (ROI) was selected from the mIF-stained tissue and matched to the corresponding ROI on the mIHC-stained section. ROIs were selected based on representative tissue morphology and staining quality, ensuring inclusion of regions with sufficient cellularity and minimal artifacts. The same ROI locations were used for both modalities to enable direct comparison of marker distribution and cell-type frequencies.

As shown in Figure 10, we found the staining to be comparable, demonstrating similar staining patterns across adjacent serial sections. In addition to comparing the staining pattern, we performed a quantification analysis to compare the cell frequencies for both types of staining across adjacent ROIs. The correlation analysis between cell frequencies for each marker in mIHC versus mIF showed significant positive correlations for all markers (Fig. 11). Despite the lack of exact cell-level correspondence between sections, consistent staining patterns were observed across matched regions of interest, supporting the accuracy of chromogen separation and marker identification. However, this approach does not allow for direct calculation of sensitivity or specificity at the single-cell level.

**Figure 10.**
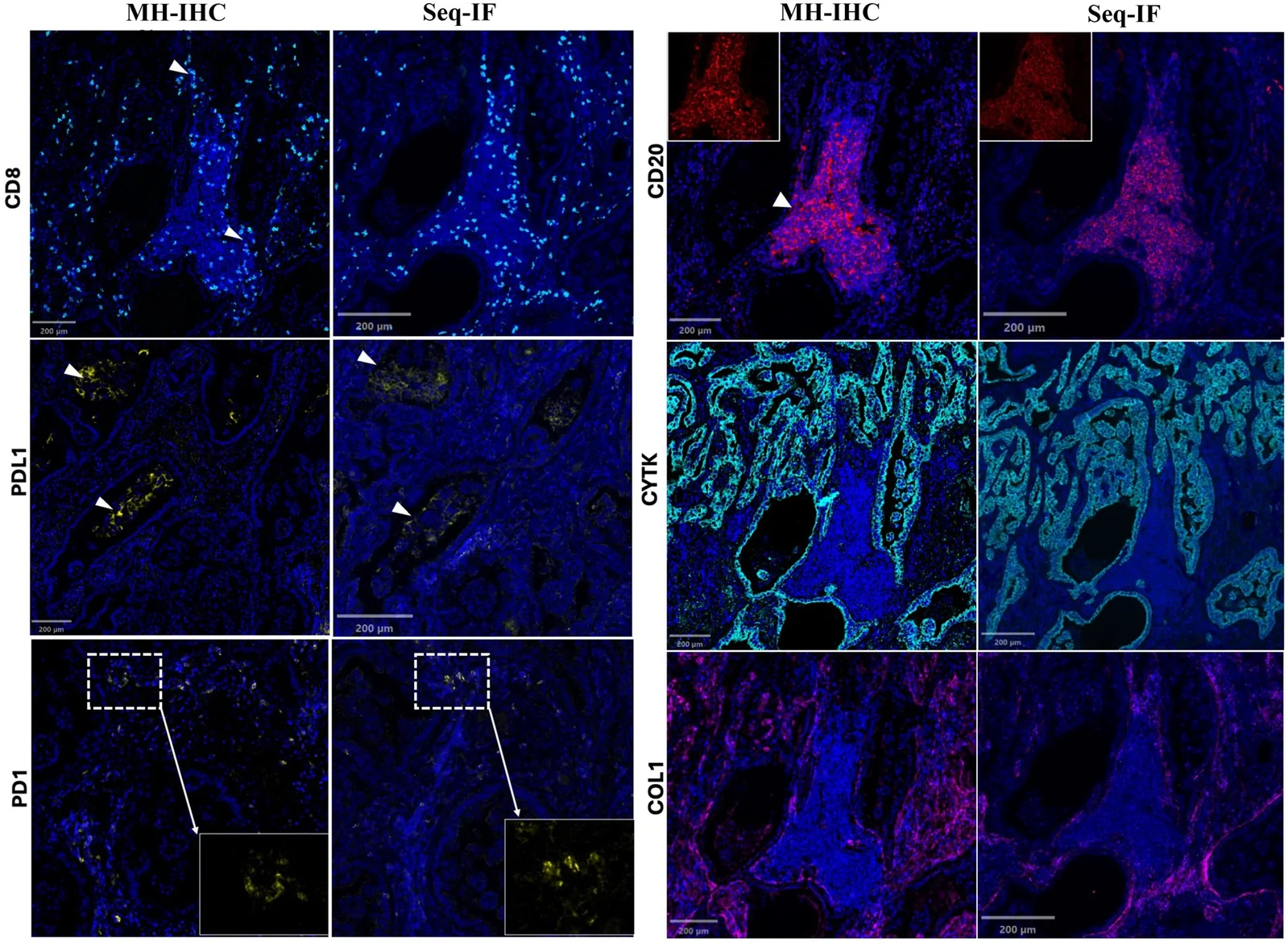
Microphotographs of representative examples of staining for CD8, PD1, PDL1, CD20, cytokeratin, and collagen1. Multiplex chromogenic IHC are shown on the left along with its corresponding adjacent section image stained with mIF (right). White arrows indicate positive staining. Antibodies stained using traditional chromogenic IHC and mIF show similar patterns of expression. Scale bars = 200 µm.

**Figure 11.**
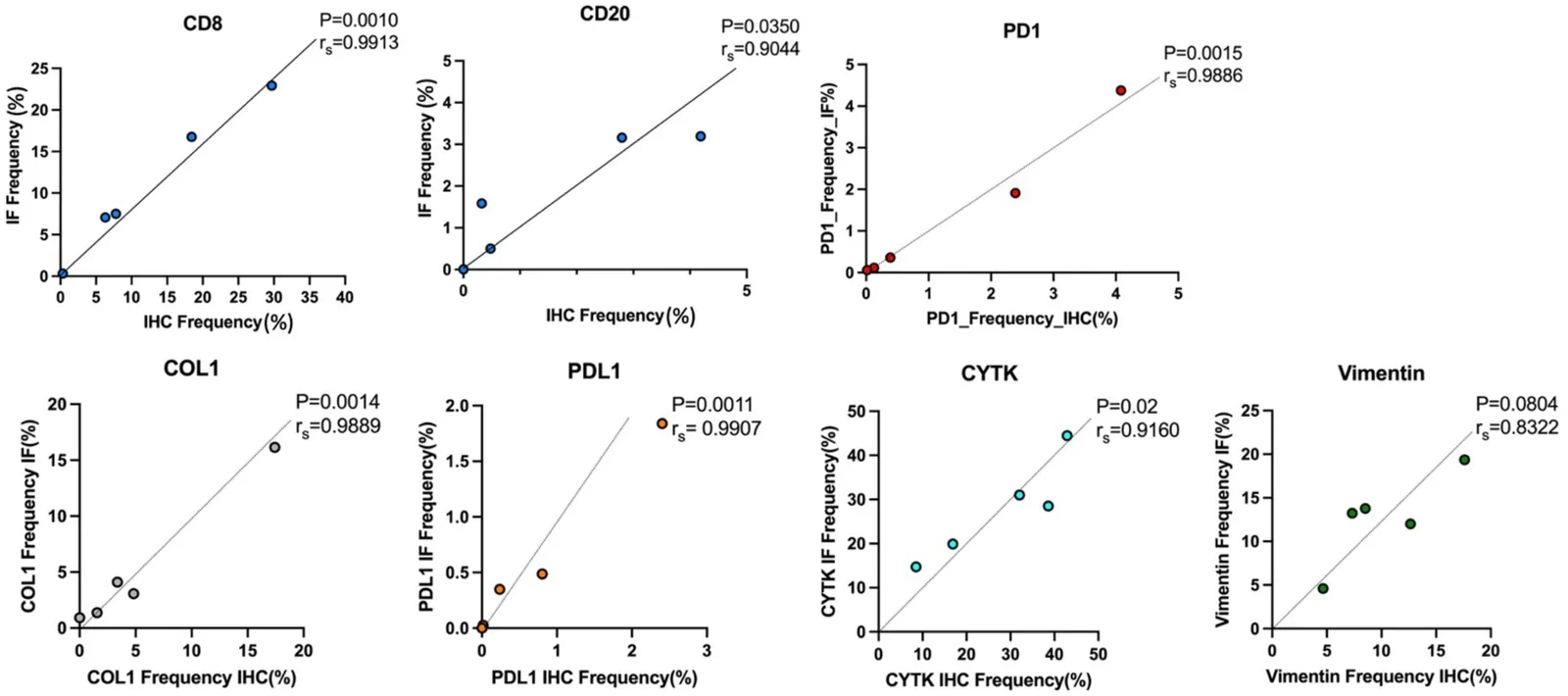
Correlation plots represent the correlation between the frequency of positive cells for each marker in multiplex chromogenic IHC versus mIF. Correlation analysis for each marker showed significant positive correlations with *P* < 0.05. Data are Pearson correlation values with *P* < 0.05.

### MH-IHC reveals cell frequencies that correlate with recurrence in eNSCLC

As a proof of principle, we applied the MH-IHC protocol to assess and quantify the different cell populations and immune–tumor cell interactions present in the TME in eNSCLC. Particularly, to determine whether certain immune cell types and mean cell neighbor frequencies present in the TME correlate with recurrence and response to immune therapy in eNSCLC.

This IHC based study enrolled 18 thoracic surgery clinic patients (6 recurrent versus 12 nonrecurrent) with confirmed eNSCLC scheduled for pulmonary resection. Following surgery, histopathologic synoptic reporting was conducted and FFPE tissue slides were subjected to the MH-IHC protocol, stained with 12 antibody markers on two adjacent serial sections, assessing key T cell populations of interest in the anti-tumor response. The markers used on each section and the rationale for their selection are shown in Table S3. The first tissue slide was stained with only immune cell markers (mIHC) to achieve 7 markers on a single tissue section, while its adjacent serial section was stained with extracellular component markers (for which accurate individual cell alignment is less critical). To minimize the impact of any cell-cell alignment errors, markers requiring precise cellular colocalization (e.g. immune phenotyping markers) were acquired from the same section, while markers derived from adjacent serial sections were selected to represent broader structural or stromal features. Combining the two, a total of 12 markers were stained on the 2 serial tissue sections.

To allow for better visualization of the various markers, concentration images representing individual marker channels obtained after unmixing are inverted to generate fluorescent-like images and each layer is assigned a unique color to distinguish the many markers in an overlaid image, particularly in cases of colocalized markers (Fig. 12).

**Figure 12.**
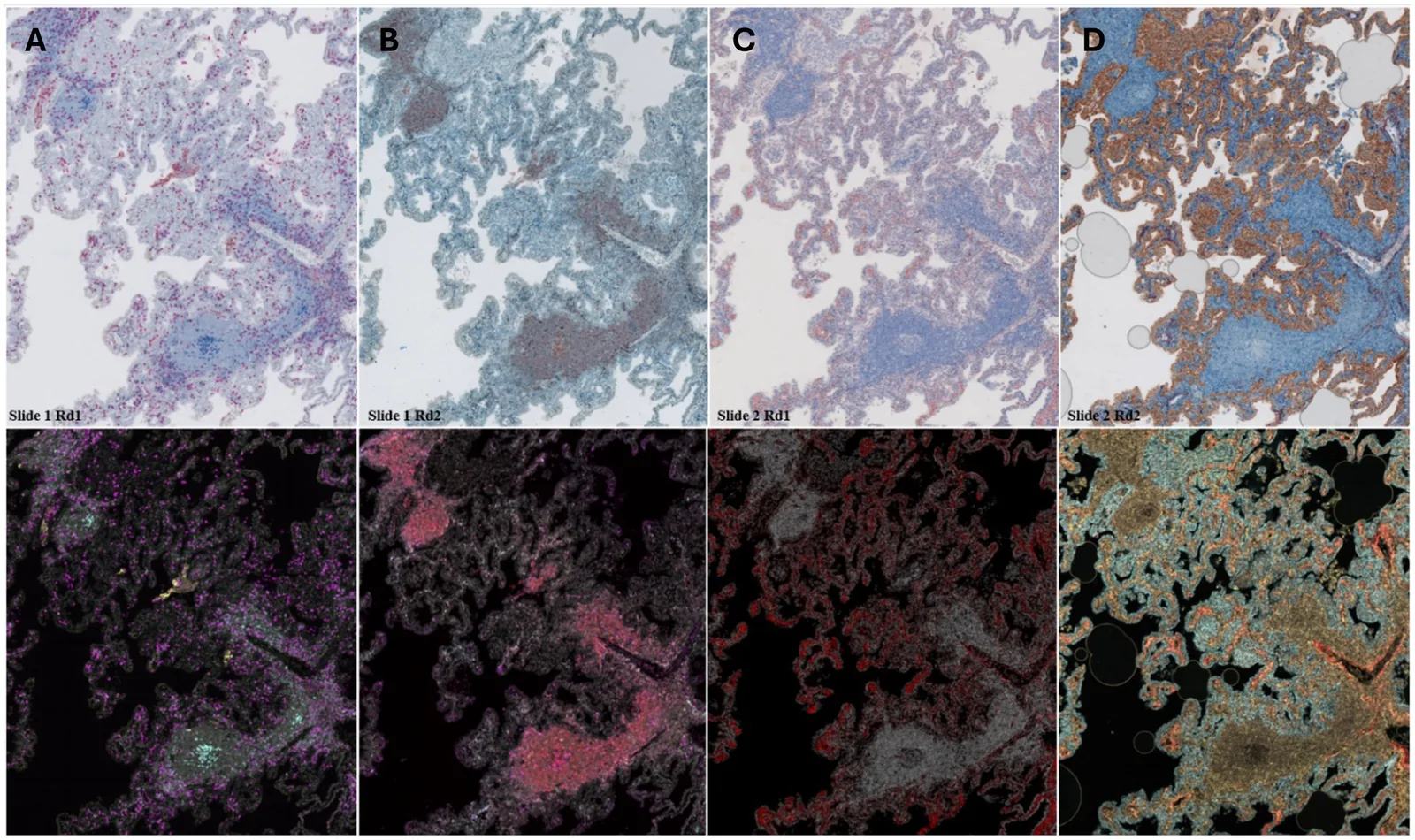
Adjacent FFPE lung tissue slides stained with the MH-IHC protocol and their individual inverted (unmixed concentrations) image generated for better visualization of the multiple markers combined in each round. (A) FFPE lung tissue slide stained in the first round with PD1, PDL1, and CD8 followed by hematoxylin counterstain. (B) FFPE lung tissue slide stained in a second round of IHC with CD20, FOXP3, CAS3, and Ki-67 followed by hematoxylin counterstain. (C) Adjacent FFPE lung tissue slide stained in a first round of IHC with collagen1 and hematoxylin counterstain. (D) Adjacent FFPE lung tissue slide stained in a second round of IHC with vimentin, pan-cytokeratin, CD31, and elastin followed by hematoxylin counterstain. Scale bars = 500 µm.

In Figure 13, we can see the results of the individual marker labels from a single tissue section after unmixing and alignment. Figure S3 shows the result for the serial section. Alignment of serial and same-slide multiple staining rounds sections, segmentation, and phenotyping was all done in Visiopharm (Fig. 14).

**Figure 13.**
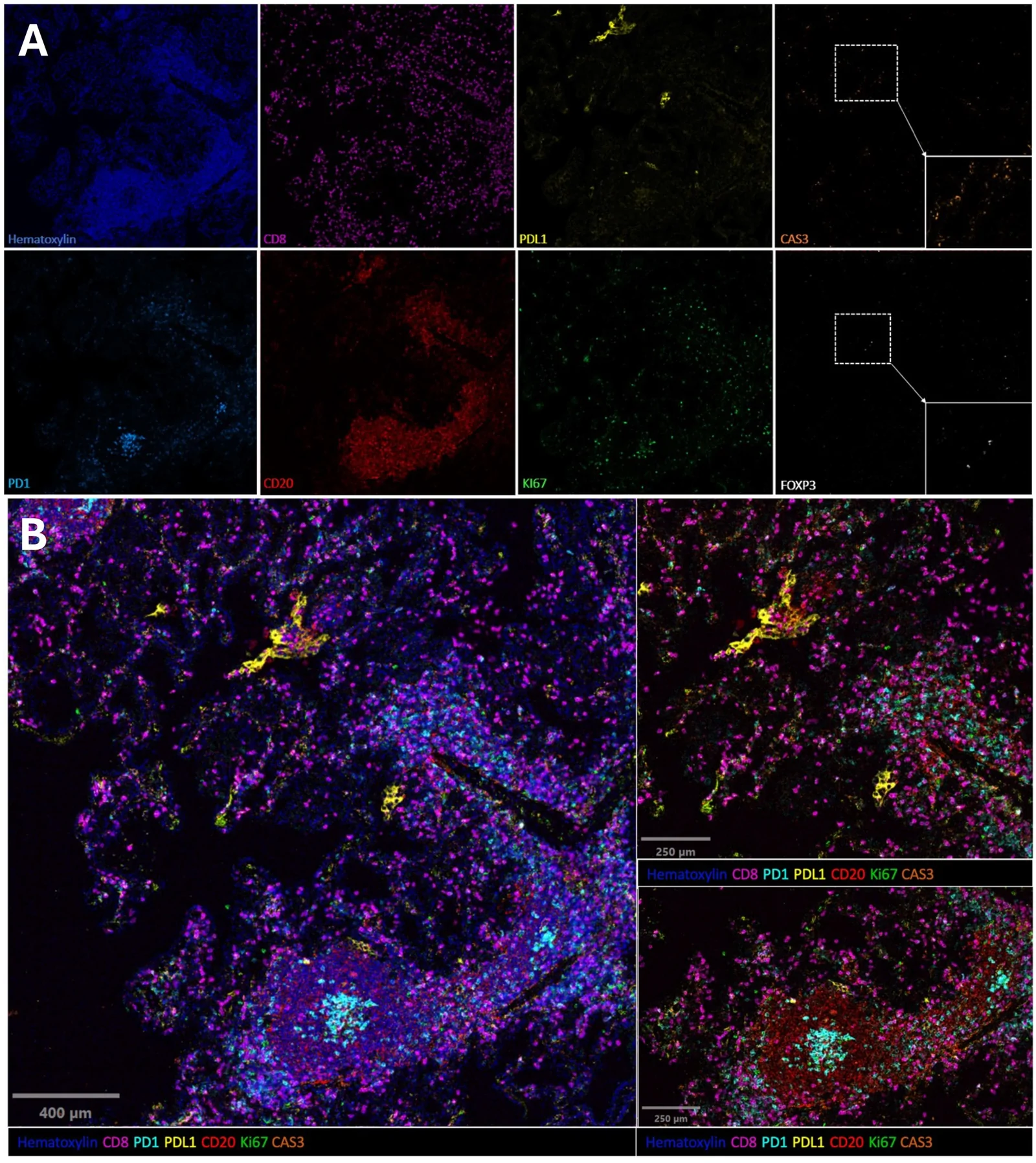
(A) Single antibody images obtained from slide 1 FFPE lung tissue after application of the mIHC. Hyperspectral workflow over 2 rounds, images are unmixed and inverted to resemble fluorescent-like images for better visualization. (B) Multiplex images with all channels aligned.

**Figure 14.**
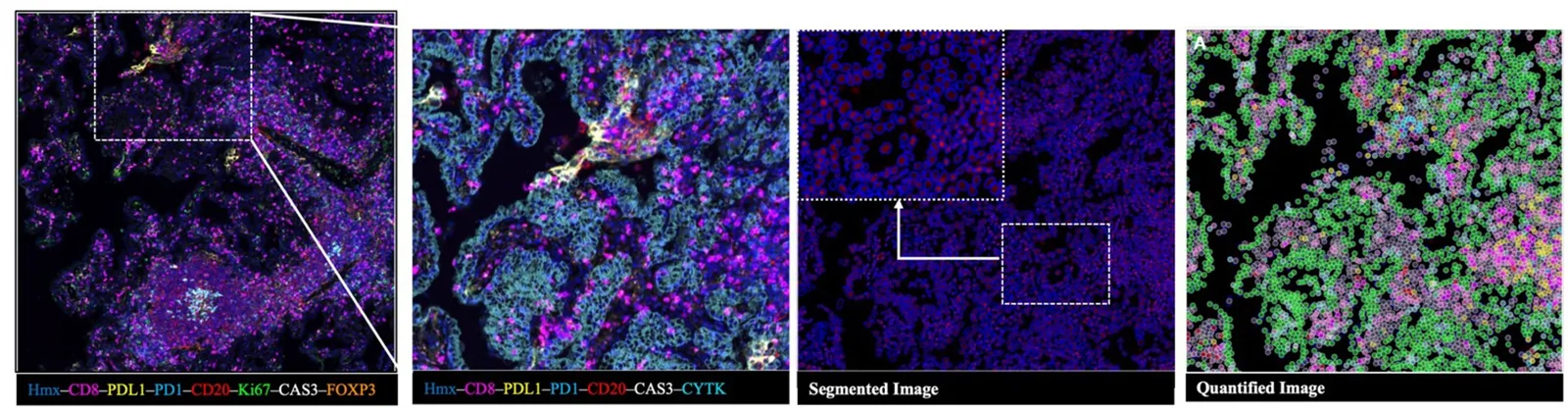
Multiplex image analysis workflow. After segmentation, using the Visiopharm Phenoplex workflow, thresholding is done for each individual marker to assess all possible marker combinations present in the tissue. Scale bars = 250 µm.

MH-IHC preliminary analyses revealed that several marker frequencies and cell–cell interactions were associated with recurrence in eNSCLC. Individual cell expression levels for the 12 markers in the biomarker panel were used to define distinct immune cell subsets and states, and cell–neighbor information was extracted for every segmented cell in the image. Patients who experienced recurrence exhibited a significantly higher proportion of cytotoxic T cells (CD8⁺), and the frequency of CD8⁺ cells neighboring one another correlated with recurrence (*P* = 0.005) (Fig. 15). In addition, CD8⁺ T cells neighboring CYTK⁺ cells were significantly more frequent in recurrence cases (*P* = 0.0203), suggesting that increased clustering of cytotoxic T cells and their proximity to malignant (CYTK⁺) cells correlates with recurrence in eNSCLC.

**Figure 15.**
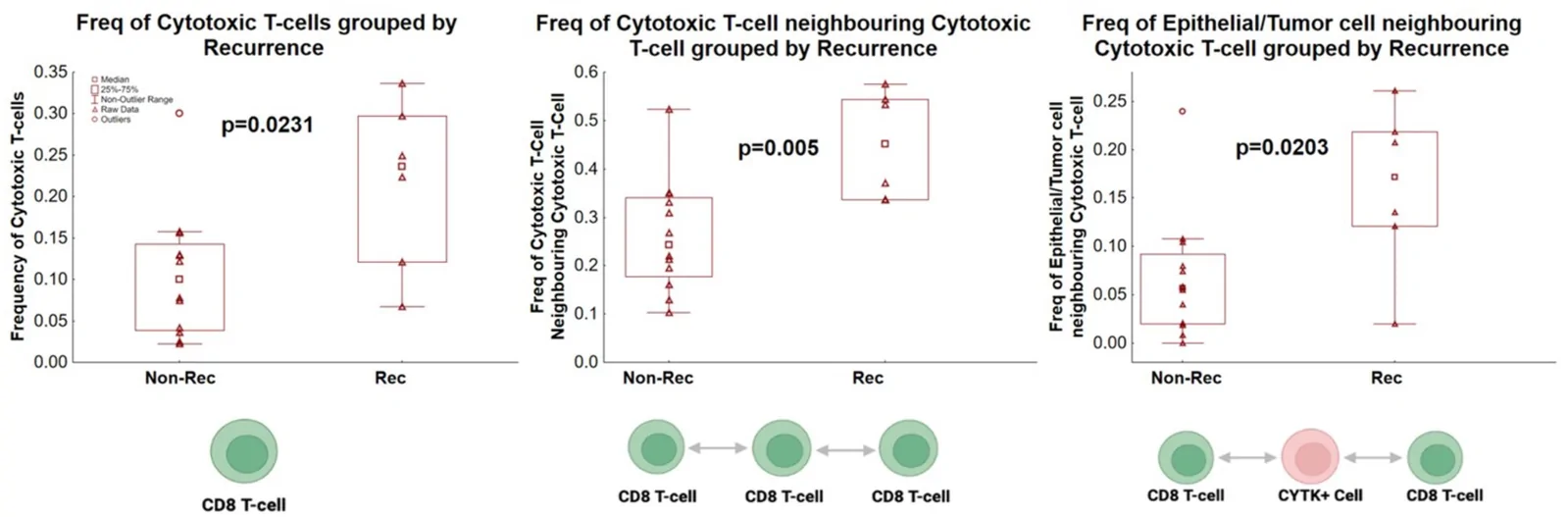
Frequencies of CD8^+^ cells, CD8^+^ neighboring CD8^+^ cells, and CYTK^+^ cells neighboring CD8^+^ cells were compared between recurrent and nonrecurrent. In all analyses, *P* < 0.05 was considered significant.

Upon further analysis, we observed that CD8⁺PD-1⁺ T cells were enriched in patients with recurrence, and the frequency of CD8⁺PD-1⁺ T cells neighboring CYTK⁺ cells was also significantly higher (Fig. 16). We next evaluated whether the expression and spatial distribution of immune cell subsets were associated with recurrence-free survival (RFS). Patients were stratified into high versus low groups based on the median value for each parameter, and Kaplan-Meier survival analyses revealed significant differences in RFS across several markers. Given the small cohort size and limited number of observed events, survival distributions between groups were compared using the 2-sample Cox *F* test implemented in Statistica (StatSoft, version 14.0.0.15; TIBCO Software). Several immune parameters were associated with RFS, including the frequency of CD8⁺PD-1⁺ T cells neighboring CYTK⁺ cells (Fig. 16). Specifically, patients with higher infiltration of CD8⁺PD-1⁺ T cells adjacent to CYTK⁺ cells exhibited significantly shorter RFS compared with those with low levels of such interactions.

**Figure 16.**
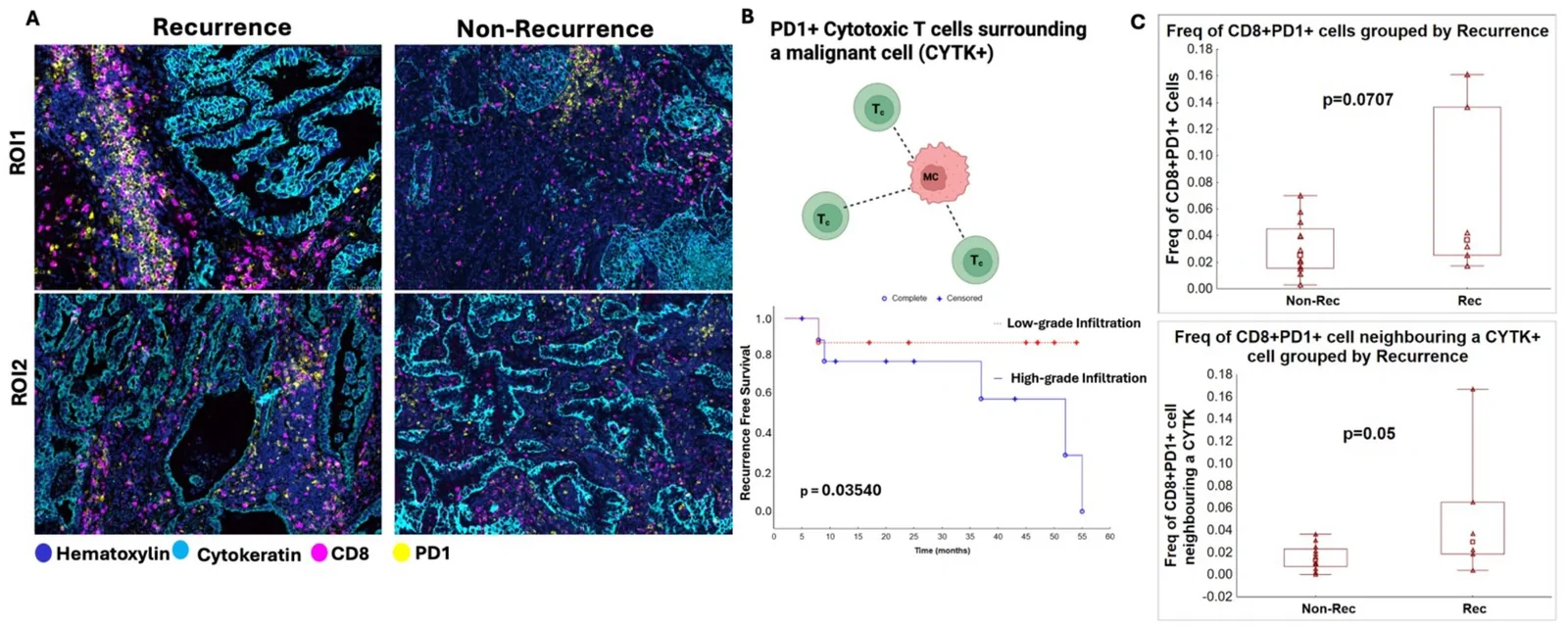
(A) Representative images of CD8, PD1, and CYTK expression in recurrence versus nonrecurrence ROIs. (B) illustration of PD1^+^ cytotoxic T cell neighboring a CYTK^+^ cell (malignant cell), The Kaplan-Meier curve illustrates the duration of overall RFS according to density of CD8^+^PD1^+^ cell neighboring another a malignant cell. Red lines represent low cell densities (or low expression) and blue lines represent high densities (or high expression). A Cox *F* test *P* < 0.05 was considered significant. (C) Box plots comparing the frequency of CD8^+^PD1^+^ T cells in recurrence versus nonrecurrence patients. Statistical differences were tested using 1-way analysis of variance; *P* values are indicated.

Taken together, these patterns suggest that the presence of cytotoxic (CD8⁺) T cells alone in recurrent tumors does not necessarily translate into effective antitumor immunity. While we observe an increased infiltration of cytotoxic T cell in recurrent tumors, many expressed PD1 on their surface, representing a population of cytotoxic T cells that have been activated and thus demonstrate an exhausted cytotoxic T cell.^23^^,^^24^ These exhausted T cells maintain tumor recognition but are functionally impaired, characterized by expression of co-inhibitory receptors and reduced production of effector cytokines essential for tumor clearance.^25^ Additionally, the increased frequency of exhausted T cells near tumor/malignant cells (CYTK^+^) indicates that immune recognition of the tumor occurs, but its response is suppressed through checkpoint signaling, allowing tumor cells to evade immune clearance.

Our findings are consistent with prior studies in treatment-naïve eNSCLC, which demonstrated that CD8⁺PD-1⁺ T cells are enriched in patients who recur, that higher frequencies of CD8⁺PD-1⁺ T cells predict worse outcomes, and that their closer proximity to CK⁺ tumor cells correlates with significantly shorter RFS and overall survival.^26^^,^^27^ These observations highlight that while CD8⁺ infiltration is abundant in recurrent tumors, this population may represent exhausted CD8⁺PD-1⁺ T cells rather than functional effectors, underscoring the prognostic importance of spatial immune metrics beyond simple cell density. This supports a broader view that recurrence does not result from a lack of immune infiltration, but rather results from ineffective or dysregulated immune engagement. Indeed, prior work has shown that CD8⁺ T cells can accumulate in clusters within the tumor microenvironment while remaining functionally exhausted, marked by checkpoint receptor expression and impaired cytotoxic activity.^28^ Similarly, increased contact between CD8⁺PD-1⁺ T cells and CYTK⁺ tumor or epithelial cells in recurrent cases may represent immune pressure that is counterbalanced by tumor-driven immune evasion, consistent with the immunoediting framework.^29^ Collectively, these results suggest that tumor cells actively induce inhibitory receptor expression on infiltrating T cells, thereby suppressing their cytotoxic activity and weakening the host antitumor response.

Overall, these results suggest that both the abundance and spatial organization of specific immune cell subsets contribute to shaping the antitumor response and clinical outcomes in eNSCLC. They emphasize the importance of assessing not only immune cell frequencies, but also spatial neighborhoods. Because this study was conducted in a relatively small patient cohort, the results should be interpreted as indicative trends rather than definitive associations. While the observed differences reached statistical significance, the limited sample size reduces the robustness of these findings. Although further validation in larger patient cohorts is needed, these findings demonstrate that MH-IHC offers a sample-efficient approach for screening and validating comprehensive prognostic panels, as well as identifying novel prognostic markers by evaluating cell–cell colocalization and immune neighborhoods.

## Discussion

With the advent of personalized cancer therapies, evaluation of both the genomic landscape and immune composition of the TME has become increasingly critical. Cell-cell communication within the TME reflects a crucial process in antitumor immunity and further investigation of the spatial organization of tumor-immune cells is essential in understanding how the TME influences tumor development, disease progression, and treatment response.^30^^,^^31^

Here, we describe a rapid and cost-effective absorption-based mIHC approach that enables analysis of complex immune cell populations and cell phenotypes within a single slide across the entire slide. MH-IHC utilizes labeling and imaging techniques that are widely available, limiting the need for additional (frequently expensive) instrumentation. Although the results were statistically significant, the small sample size limits the ability to draw strong inferences about the statistical significance of the observed results and thus findings are best interpreted as preliminary trends that warrant further validation in larger patient cohorts.

Several methods for high-dimensional analysis of tissue lesions on a single tissue slide have been described in the literature, ranging from brightfield IHC techniques to fluorescence staining. For chromogenic absorption-based IHC, most mIHC methods mentioned are achieved through multiple rounds of staining of a single marker labeled with AEC over several rounds.^13^ Although such techniques enable the detection of multiple biomarkers on a single slide, they require subjecting the tissue to several rounds of staining/destaining cycles, which can be time-consuming and may compromise tissue integrity when attempting to stain several markers at once.

Other techniques that utilize multispectral imaging of newly optimized chromogens with relatively narrow absorbance spectra can achieve multiplexing of approximately 4 or 5 markers by minimizing spectral overlap, as demonstrated by Morrison et al.^15^ These approaches rely on selecting illumination wavelengths matched to chromogen absorbance bands and perform spectral unmixing using predefined spectral profiles.

In contrast, the method presented here works with standard chromogens commonly employed in brightfield IHC, such as AEC, Ferangi Blue, DAB, and Fast Red, and can be readily applied across multiple rounds through iterative destaining and restaining of the same section.

Other multiplex platforms that utilize fluorescence staining also exist, many of these approaches are able to achieve larger numbers of biomarkers on a single slide through iterative cycles of staining, imaging, and fluorophore inactivation or removal. For instance, the Lunaphore COMET 1.0 platform stains 2 markers per cycle utilizing a microscope-integrated microfluidic system and can achieve over 40 markers on a single slide over an area 1.2 cm by 1.2 cm.^32^ Similarly, Leica’s Cell DIVE system enables iterative staining of 4 markers per round, followed by imaging (of the entire slide) and repeated cycling, allowing the analysis of panels exceeding 60 markers.^33^ While these technologies provide high-dimensional spatial information as they are capable of labeling several biomarkers on a single tissue slide, a major limitation is that they require the use of costly reagents and fluorescent dyes that are light-sensitive and can only be imaged with specific imaging scanners to overcome or limit any issues of spectral overlap (AKOYA-OPAL, Lunaphore, Ultivue, Cell Dive).^34^^,^^35^

In contrast, a key advantage of MH-IHC is its practicality and accessibility. The workflow can be implemented in standard pathology laboratories using routine chromogenic IHC staining platforms, with the only additional requirement being access to a high-throughput scanner (e.g. Akoya PhenoImager or Mantra system, now part of Quanterix).^36^^,^^37^ This makes the technique broadly scalable and suitable for both research and translational applications. In terms of processing time, each staining cycle requires approximately 6 to 7 h for 3 or 4 antibodies. Automated immunostaining platforms enable batch processing of up to ∼50 slides simultaneously, allowing multiple slides to be stained in parallel. Consequently, overall throughput is primarily limited by image acquisition between staining rounds rather than the staining process itself.

Taken together, MH-IHC offers a cost-effective and easily applicable alternative for laboratories seeking to extract multiplexed spatial information without the need for specialized equipment that can be used to interrogate the entire area of a slide.

Over the past few years, the availability of multiple spatial analysis tools capable of analyzing the distribution of cells within the TME has increased significantly.^38–40^ MH-IHC overcomes the limitations of conventional IHC methods as it requires minimal tissue sample availability, enhancing the depth of biological insight gained from a single tissue section. This is essential in clinical research in which sample availability may be limited. In addition, it significantly reduces the time required to stain for multiple markers as it is capable of achieving up to 7 markers in only 2 rounds. Although our protocol has been validated and optimized over several tissue samples, any change or adjustment in the protocol does require further validation. We do recommend staining for weaker markers in the primary round, to avoid any possible antigenicity loss that may occur with certain markers. In addition, when optimizing a new panel of markers, trying different staining sequences to find the optimal configuration for each antibody is preferred.

In summary, our approach offers an absorption-based multiplexed IHC method for high-dimensional tissue analysis, maximizing the spatial information obtained from a single tissue section. This method can potentially be integrated and implemented in most pathology laboratories, as the resources and equipment required are widely accessible and less costly than their fluorescence analogues.

## Conclusion

We have developed a chromogenic mIHC technique capable of staining up to 14 markers across 2 adjacent tissue sections (7 on each tissue section), allowing us to characterize the tissue cell phenotypes and the spatial distribution of cells within the TME. MH-IHC can be used to quantify the spatial interactions within the TME, providing biological insight that cannot be acquired from cell density information alone. In summary, we describe a multiplex brightfield technique that can be used on any FFPE tissue to maximize the quantitative and spatial information that can be obtained from valuable tissue samples.

## Supplementary Material

vlag054_Supplementary_Data

## Data Availability

The data underlying this article are available in the article and in its online supplementary material.
