# Supplementary material for: Multiplex hyperspectral IHC: A 12-plex assay for high-dimensional analysis of the tumor microenvironment
Source: Immunohorizons. 2026 Oct 15;10(10):vlag054. doi: 10.1093/immhor/vlag054 (PMC13672375; doi:10.1093/immhor/vlag054)
Supplement: Supplementary file 1 [file vlag054_supplementary_data.docx]

**Supplementary Material**

**Figure S1:** Image Alignment

**Figure S1.** Image alignment for adjacent sections is done in Visiopharm using the Tissue Align module, which consists of an alignment at low magnification that handles rotation, translation and any deformations in the tissue and alignment at high-magnification that’ builds on aligning local regions. In the case where further alignment is needed, we can improve it using pins designating landmarks (homologous tissue structures/features) in the corresponding images. We attempted to use immune-cell markers to identify cell types all within one section, while its adjacent section contains markers for extracellular components which generally have extensive expression limiting the degree of need for cell component level alignment between the two sections. **A.** Hematoxylin from section 1 aligned with hematoxylin from its adjacent section. **B.** Hematoxylin from section 1 aligned with Elastin from its adjacent section. **C.** Hematoxylin from section 1 aligned with Collagen1 from its adjacent section.

For same slide alignment, inverted concentration images obtained after unmixing from round 1 and 2 for each slide are loaded and the automatic alignment is selected. After aligning the 2 images, images are exported as two separate images and must be combined in ImageJ into a single ome.tiff file. Once images from the same tissue sections have been aligned, OME.tiff files obtained after alignment of each slide are loaded and images from adjacent sections are aligned similarly. If the result of the automatic alignment of adjacent sections is not satisfactory, it was improved though the use of an observer designating landmarks (homologous tissue structures/features) in the corresponding images.

**Figure S2:** Hyperspectral Scanning and LED System

**Figure S2**.**LED Hardware**. Image of the LED condenser in the hyperspectral system, the condenser has been replaced with a 7-LED illumination light source. The setup includes two Genuino Uno (ATmega328) microcontrollers, seven independent 150–1500 mA buck-regulator LED drivers. Each LED driver corresponds to one of seven discrete wavelengths (407, 460, 523, 590, 623, 660 and 740 nm) and powers an LED embedded within the condenser. Although the hyperspectral imaging system was developed in-house, comparable systems are commercially available.

**Table S1: Patient demographics and clinical characteristics.**

| **Characteristics** | **Non-recurrence** | |  | **Recurrence** | |
| --- | --- | --- | --- | --- | --- |
|  | **n** | **%** |  | **n** | **%** |
|  | 12 | 67% |  | 6 | 33% |
| **Histology** |  |  |  |  |  |
| Adenocarcinoma | 9 | 75% |  | 5 | 83% |
| Squamous cell carcinoma | 3 | 25% |  | 1 | 17% |
| **Age (years)** | 72.5 |  |  | 71 |  |
| **Sex** |  |  |  |  |  |
| Female | 9 | 75% |  | 6 | 100% |
| Male | 3 | 25% |  | 0 | 0% |
| **Race/Ethnicity** |  |  |  |  |  |
| White | 9 | 75% |  | 5 | 83% |
| Mixed/Other | 1 | 8% |  | 0 | 0% |
| Asian | 2 | 17% |  | 1 | 17% |
| **Smoking history** |  |  |  |  |  |
| Current | 3 | 25% |  | 1 | 17% |
| Former | 4 | 33% |  | 3 | 50% |
| Never | 5 | 42% |  | 2 | 33% |
| **Pathologic stage** |  |  |  |  |  |
| IA | 7 | 58% |  | 1 | 17% |
| IB | 1 | 8% |  | 0 | 0% |
| IIB | 3 | 25% |  | 3 | 50% |
| IIA | 1 | 8% |  | 1 | 17% |
| IIIA | 0 | 0% |  | 1 | 17% |
| **Pathologic T** |  |  |  |  |  |
| T1a | 2 | 17% |  | 1 | 17% |
| T1b | 5 | 42% |  | 1 | 17% |
| T1c | 3 | 25% |  | 0 | 0% |
| T2a | 1 | 8% |  | 2 | 33% |
| T3 | 1 | 8% |  | 2 | 33% |
| **Pathologic N** |  |  |  |  |  |
| N0 | 10 | 83% |  | 3 | 50% |
| N1–N3 | 2 | 17% |  | 3 | 50% |

**Table S1.** Clinicopathologic information of the patients included in the study.

**Table S2: List of primary antibodies and working dilutions.**

| **Antibody** | **Manufacturer** | **Catalog No.** | **Host** | **Dilution** |
| --- | --- | --- | --- | --- |
| **PD1** | Abcam | ab52587 | Mouse | 1:100 |
| **CD8** | Abcam | ab17147 | Mouse | 1:100 |
| **PDL1** | Abcam | ab228462 | Rabbit | 1:100 |
| **CD20** | Abcam | ab78237 | Rabbit | 1:100 |
| **FOXP3** | Abcam | ab99963 | Rabbit | 1:50 |
| **KI67** | SantaCruz | sc23900 | Mouse | 1:50 |
| **CASP3** | Thermofisher | 3CSP01 | Mouse | 1:200 |
| **Pan-cytokeratin** | Abcam | ab80826 | Mouse | 1:100 |
| **Elastin** | Abcam | ab23747 | Rabbit | 1:1000 |
| **Collagen** | Abcam | ab34710 | Rabbit | 1:1000 |
| **CD31** | Abcam | ab28364 | Rabbit | 1:50 |
| **Vimentin** | Abcam | ab92547 | Rabbit | 1:1000 |

**Table S2.** List of primary antibodies used in this study, including antibody manufacturer, catalog number, host, and working dilution.

**Table S3: Summary of antibodies used for multiplexed brightfield immunohistochemistry and their biological rationale**

| **Antibody** | **Rationale for Biomarker Selection** |
| --- | --- |
| **PD1** | T-cell marker; expressed on the surface of activated T-cells |
| **PDL1** | Tumour cells, attachment to PD1 blocks anti-tumour immune response |
| **CD8** | Cytotoxic T cells |
| **FOXP3** | Regulatory T cell markers (Treg), prognostic in human cancers |
| **CD20** | B cell marker, thought to potentiate anti-tumor immunity |
| **Caspase-3 (CAS-3)** | Apoptosis marker, cell death |
| **Vimentin (VIM)** | Epithelial mesenchymal transition marker |
| **Ki67** | Cellular proliferation, assess patient survival and cancer progression |
| **CD31** | Endothelial cell marker, tumour angiogenesis |
| **Collagen1** | Fibrosis, main component of ECM protein provides resistance and support to tissues |
| **Elastin** | Fibrosis; provides the characteristic elasticity properties in many soft tissues |
| **Cytokeratin (CK)** | Epithelial cell |

**Table S3.** Summary of primary antibodies included in the multiplexed panel and their corresponding biological relevance.

**Figure S3:** MH-IHC applied on adjacent Lung section

**Figure S3.** A. Single antibody images obtained from adjacent FFPE lung tissue after application of the mIHC Hyperspectral workflow over 2 rounds, images are unmixed and inverted to resemble fluorescent-like images for better visualization. B. Multiplex image with all channels aligned.

**A**

**B**
